# Deletion mutagenesis of large areas in *Plasmodium falciparum *genes: a comparative study

**DOI:** 10.1186/1475-2875-6-64

**Published:** 2007-05-22

**Authors:** Marni Williams, Abraham I Louw, Lyn-Marie Birkholtz

**Affiliations:** 1 Department of Biochemistry, School of Biological Sciences, Faculty of Natural and Agricultural Sciences, University of Pretoria, Pretoria, 0002, South Africa

## Abstract

**Background:**

The increasing emergence of *Plasmodium falciparum *parasites resistant to most of the cost-effective drugs has necessitated the identification of novel leads and drug targets. Parasite-specific inserts in enzymes that are essential for the differentiation and proliferation of malarial parasites have received considerable interest since it distinguishes these proteins from their human counterparts. The functions of these inserts, which include mediations of protein activities or protein-protein interactions, are being investigated by several strategies including deletion mutagenesis. A comparative study of five widely used PCR-based mutagenesis methods identified a modified inverse PCR method as particularly suitable for the deletion of large areas (>100 bp) in malaria parasite genes.

**Methods:**

The restriction enzyme-mediated inverse PCR method described here incorporates unique restriction enzyme sites at the 5'-ends of inverse tail-to-tail primers. The entire gene-containing vector is amplified except the desired region to be deleted and cloned using the unique restriction sites to increase ligation efficiency. This method was compared in its efficiency to delete a ~400 bp parasite-specific insert in malarial *S*-adenosylmethionine decarboxylase/ornithine decarboxylase (PfAdoMetDC/ODC) to existing PCR-based site-directed deletion mutagenesis methods including the QuickChange™ site-directed mutagenesis, ExSite™, overlapping primer and inverse PCR. In addition, the modified method was applied in the deletion of a >600 bp parasite-specific insert in another malarial gene, pyridoxal kinase (PfPdxK).

**Results:**

The modified and optimized restriction enzyme-mediated inverse PCR method resulted in 80% compared to 40% deletion mutagenesis efficiency of the overlapping primer method in the deletion of a large area (411 bp) from a large malaria gene (PfAdoMetDC/ODC, gene size 4257 bp). In contrast, deletion mutagenesis methods such as the well-known QuickChange™ site-directed mutagenesis, ExSite™ and inverse PCR methods produced insignificant results. A 100% mutagenesis efficiency was obtained with the restriction enzyme-mediated inverse PCR method to delete 618 bp from a smaller gene (PfPdxK, gene size 1536 bp).

**Conclusion:**

An efficient method was developed for the deletion of large areas (>100 bp) in significantly sized genes such as those of the A+T-rich *P. falciparum *genome.

## Background

Specialized organisms like *Plasmodium falciparum *have unique adaptations, which include generally larger protein sizes compared to orthologues due to bifunctional arrangements of proteins and the presence of parasite-specific inserts [1]. In general, these inserts are species-specific, rapidly diverging, non-globular regions containing low-complexity areas consisting of mainly Lys and Asn residues that form flexible prion-like domains extending from the protein core [2,3]. Up to 90% of *P. falciparum *proteins contain at least one low-complexity region, which may co-localize with parasite-specific inserts. These proteins are also up to 50% longer compared to their yeast counterparts [4,5]. The exact evolutionary origin and functional advantages of these inserts remain elusive. It has, however, been proposed by Karlin *et al *[6] that these inserted regions are adaptive as they seem to promote protein-protein interactions and mRNA stability. For example, in *P. falciparum *it has been demonstrated that stabilization of interdomain interactions of the bifunctional malarial drug target, dihydrofolate reductase (DHFR)/thymidylate synthase (TS), is mediated via an essential parasite-specific insert [7,8]. DHFR/TS also regulates its own translation by binding to cognate mRNA [9]. Some parasite-specific inserts have been implicated in malaria pathogenesis due to an increase in the antigen diversity and resultant incomplete immune response of the human host to *P. falciparum *[4].

**Figure 1:**
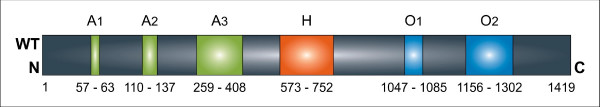
**Parasite-specific inserts in the bifunctional AdoMetDC/ODC protein**. The figure shows the wild type *P. falciparum *AdoMetDC/ODC protein with the positions and residue numbers of the parasite-specific inserts indicated (A_1_, A_2_, A_3_, H, O_1_, O_2_) [11-13]. Inserts in the AdoMetDC domain are indicated in green (A_1_, A_2_, A_3_), the hinge area in orange (H), and the ODC inserts are shown in blue (O_1_, O_2_). The N- and C-terminals are also indicated.

The bifunctional *P. falciparum S*-adenosylmethionine decarboxylase/ornithine decarboxylase (PfAdoMetDC/ODC) regulates the synthesis of polyamines, essential molecules for DNA and RNA stabilization [10]. In addition to its unique bifunctional nature, the protein contains six parasite-specific inserts of up to 411 bp (Figure 1) [11-13]. Analysis of the structure-activity relationships indicated that these inserts are important for protein activity of the respective decarboxylase domains and act as mediators of protein-protein interactions in the bifunctional protein complex [14].

**Figure 2:**
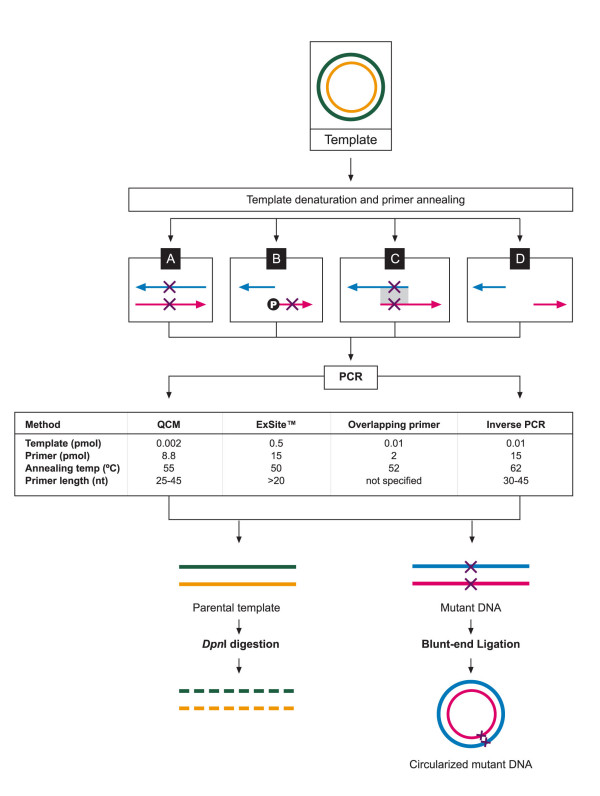
**Overview of four currently used deletion mutagenesis methods**. A) QuickChange™ site-directed mutagenesis method, B) ExSite™ site-directed mutagenesis method, C) Overlapping primer method, D) Inverse PCR method. The template plasmid (green and yellow, top panel) is denatured during the first step of the PCR reaction to allow the primers containing the desired mutation to anneal to the specific target sites. In the QCM method the primers completely overlap and the mutations are incorporated into both of the primers (A). The ExSite primers do not overlap at all; instead only one of the primers contains the mutation. One or both of the primers must also be phosphorylated (B). Partial overlapping at the 3' ends is characteristic of the overlapping primers. The mutation is present in both of the primers within the overlapping region (C). And finally, primers for the inverse PCR method do not overlap, but simply start at the opposite ends of the desired area to be deleted (D). The pink and blue arrows are the sense and antisense primers. Crosses on the primers represent the mutation sites and P is the single phosphorylated ExSite™ primer in B. The PCR conditions for the specific deletion of a ~400 bp parasite-specific insert from the 7.4 kb template (described here) are given in the table. The primers are extended at 68°C during which the desired mutations are incorporated and subsequently amplified. Two different linear PCR products are created during the PCR reactions, parental template and mutated DNA. The parental DNA is degraded by a *Dpn*I digestion step while the mutant DNA is circularized by blunt-end ligation. The newly formed mutant plasmids can subsequently be transformed into competent cells

Site-directed mutagenesis is an important technique used in studying protein structure-activity relationships. Non-PCR based deletion mutagenesis methods mostly use sequence-specific exonuclease-based enzymatic procedures but has the disadvantage that a single-stranded template is required [15]. Since the development of PCR, oligonucleotide-mediated site-directed deletion mutagenesis has become a technically straightforward and efficient endeavour (for review see [15,16]). Widely used PCR-based mutagenesis methods include the QuickChange™ site-directed mutagenesis (QCM) and ExSite™ methods (Stratagene), which are effective for the deletions of areas of up to 12 bp (Figure 2A and 2B, respectively) [17]. Several modifications to the QCM have been reported to be successful for deletions in large genes [18-20]. This includes a partial overlapping primer design method allowing 7 bp deletions (Figure 2C) [19] and inverse PCR methods with a maximal deletion of 102 bp (Figure 2D) [18]. However, none of these methods have been reported to be consistent in removing areas >100 bp in genes.

In this study, a restriction enzyme (RE)-mediated inverse PCR is described that successfully removes large areas in abnormally large genes (gene size ~4.3 kb). The deletion mutagenesis efficiency of this RE-mediated inverse PCR method was compared to the existing methods described above by deleting a 411 bp parasite-specific insert in the AdoMetDC domain of the bifunctional PfAdoMetDC/ODC protein. In addition, its application to delete an insert in another malarial gene was also investigated.

## Methods

All five methods described below (QCM, ExSite™, overlapping primer, inverse PCR and RE-mediated inverse PCR) used the *P. falciparum *AdoMetDC/ODC gene (gene size ~4.3 kb) cloned into a pASK-IBA3 vector (vector size ~3.1 kb; Institut für Bioanalytik, Göttingen, Germany) as template (total template size ~7.4 kb) [13]. The mutagenesis primers designed for use in the different methods are given in Table 1.

**Table 1:** Primers used for the various mutagenic protocols. The *Bam*HI restriction sites for primer pairs P3 and P4 are underlined. The P3 primer pair was used with and without *Bam*HI restriction digestion for the RE-mediated inverse PCR and inverse PCR methods, respectively.

**Primer Pair**	**Primer**	**Length (bp)**	Tm* (°C)	**Primer Sequence (5' to 3')**	**Mutagenesis method**
P1	A3consF	43	78	gctttatgatagtagtgatgctgataattataataaggaaagc	QuickChange™ site-directed method
	A3consR	43	78	gctttccttattataattatcagcatcactactatcataaagc	
P2	A3overF	49	79	gatagtagtgatgctgat ↓ aattataataaggagagctttttatataatg	Overlapping primer method [19]
	A3overR	55	80	gctttccttattataatt ↓ atcagcatcactactatcataaagctttaaattatcc	
P3	A3reF	27	73(62)	cgcggatccaattataataaggaaagc	ExSite™, inverse [18] and RE-mediated inverse PCR methods
	A3reR	34	79(69)	cgcggatccatcagcatcactactatcataaagc	
P4	PdxkF	34	78(67)	cgcggatccaatctaaattttctttgggtatgtg	RE-mediated inverse PCR method
	PdxkR	38	79(67)	cgcggatcctttccttcttaattcaagtatatttttgg	

* The Tm's were calculated according to the Stratagene formula: 81.5 + 0.41(%GC) - 675/N) or for primer pairs P3 and P4 with the Rychlik *et al. *formula: 69.3 + 0.41(%GC) - (650/N) [26] as indicated in parentheses.

↓ indicates where the deletions are made with the overlapping primers.

### QuickChange™ site-directed mutagenesis (QCM)

According to the manufacturers' recommendations, a 50 μl reaction contained 10 ng template (0.002 pmol for the 7.4 kb template used here), 125 ng of each of the primers (8.8 pmol each of A3consF and R), 1 × *Pfu *reaction buffer, 200 μM of each dNTP, and 2.5 U *Pfu *DNA polymerase (Fermentas, Burlington, Canada). The temperature cycles were as follows: incubation at 95°C for 30 sec, followed by 30 cycles of 95°C for 30 sec, 55/60°C for 1 min, 68°C for 2 min/kb and a final extension at 68°C for 2 min/kb.

### ExSite™ PCR-based site-directed mutagenesis

The PCR reaction with a final volume of 25 μl was set up as follows: 0.5 pmol template, 15 pmol of each primer (A3reF and R), 1 × *Pfu *reaction buffer, 200 μM of each dNTP, and 2.5 U *Pfu *DNA polymerase (Fermentas, Burlington, Canada). The temperature cycles were as follows: incubation at 94°C for 4 min, 50°C for 2 min, 68°C for 2 min/kb of template, followed by 18 cycles of 94°C for 1 min, 56°C for 2 min, and 68°C for 1 min/kb, followed by a final incubation at 68°C for 5 min.

### Overlapping primer method

A typical deletion mutagenesis reaction for the overlapping primer protocol with a final volume of 50 μl, contained 50 ng template (0.01 pmol for the 7.4 kb template), 2 pmol of each primer (A3overF and R), 1 × *Pfu *reaction buffer, 200 μM of each dNTP and 2 U of *Pfu *DNA polymerase (Fermentas, Burlington, Canada). The cycling parameters were 94°C for 3 min, 16 cycles of 94°C for 1 min, 52°C for 1 min and 2 min/kb at 68°C with a final extension for 1 hour at 68°C according to the method described by Zheng *et al *[19].

### Inverse PCR method

The PCR reaction set up followed the protocol as indicated by Wang *et al *[18]. A typical deletion mutagenesis reaction with a 50 μl final volume contained 50 ng template (0.01 pmol for the 7.4 kb template), 150 ng of both primers (15 pmol of A3reF and R), 1 × *Pfu *reaction buffer, 200 μM of each dNTP and 2.5 U *Pfu *DNA polymerase (Fermentas, Burlington, Canada). The temperature cycles were as follows: 95°C for 3 min, 18 cycles of 95°C for 45 sec, 62°C for 1 min and 68°C for 2 min/kb, with a final extension at 68°C for 2 min/kb. The same reaction was also repeated with the addition of 5% DMSO.

### RE-mediated inverse PCR method

For the RE-mediated inverse PCR method described here a typical deletion mutagenesis reaction with a 50 μl final volume contained 50 ng template (0.01 pmol for the 7.4 kb template), 150 ng of both primers (15 pmol each of A3reF and R), 1 × *Pfu *reaction buffer, 200 μM of each dNTP and 2.5 U *Pfu *DNA polymerase (Fermentas, Burlington, Canada). The temperature cycles were typically as follows: 95°C for 3 min, 18 cycles of 95°C for 45 sec, 56°C (Table 1) or 62°C for 1 min and 68°C for 2 min/kb, with a final extension at 68°C for 2 min/kb [18]. The abovementioned conditions were chosen for comparative purposes to the other methods. However, follow-up experiments have shown a 10^5 ^molar excess of primer:template to be optimal (template concentrations ranging from 0.01 pmol to 0.15 fmol with 15 pmol primer). In addition, 2.5 U ExTaq (TaKaRa Biomedicals, Shiga, Japan) was used on this larger template.

The effectiveness of this method in deleting a parasite-specific insert [21] in another A+T rich *P. falciparum *gene was also tested. The *P. falciparum *PdxK gene (gene size ~1.5 kb) was cloned into the same pASK-IBA3 vector (total template size ~4.6 kb) [22]. The reaction conditions as well temperature cycles were identical as given above.

### Analysis of mutagenesis products

PCR products were analysed with 1% agarose electrophoresis to determine if the correct sized mutant products were obtained. Correctly sized PCR products were subsequently treated with 10 U of *Dpn*I for 3 hrs at 37°C to remove parental templates. For the restriction-mediated inverse PCR method, 10 U *Bam*HI (Fermentas, Burlington, Canada) was additionally added to the *Dpn*I digestion in a dual compatibility buffer Tango™ (33 mM Tris-acetate pH 7.9, 10 mM Mg-acetate, 66 mM K-acetate and 0.1 mg/ml BSA). Products were purified with the HighPure PCR product purification kit (Roche Diagnostics, Mannheim, Germany) and ligated for 16 hrs with 3 U of T4 DNA Ligase (Promega, Wisconsin, USA) at 4°C. The resulting circular plasmids were transformed into electrocompetent DH5α cells (Gibco BRL, Life Technologies, USA). Five clones for each of the three different mutagenesis methods that produced PCR products were analysed with *Hind*III restriction mapping. In the wild type gene, this enzyme should cut twice resulting in three bands of ~3900, ~3100 and ~450 bp. However, in the ~400 bp deletion mutants, one of the sites is removed resulting in only two bands sized ~3900 and ~3100 bp. These same clones were thereafter analysed with nucleotide sequencing to confirm the mutagenesis results. Application of the restriction-mediated inverse PCR method on the deletion of ~600 bp from the PfPdxK gene also resulted in a PCR product. Five clones were analysed with *Eco*RI restriction mapping. The deletion removes an *Eco*RI site resulting in the linearization of only the wild type nonmutated DNA (~4.6 kb template).

## Results

**Figure 3:**
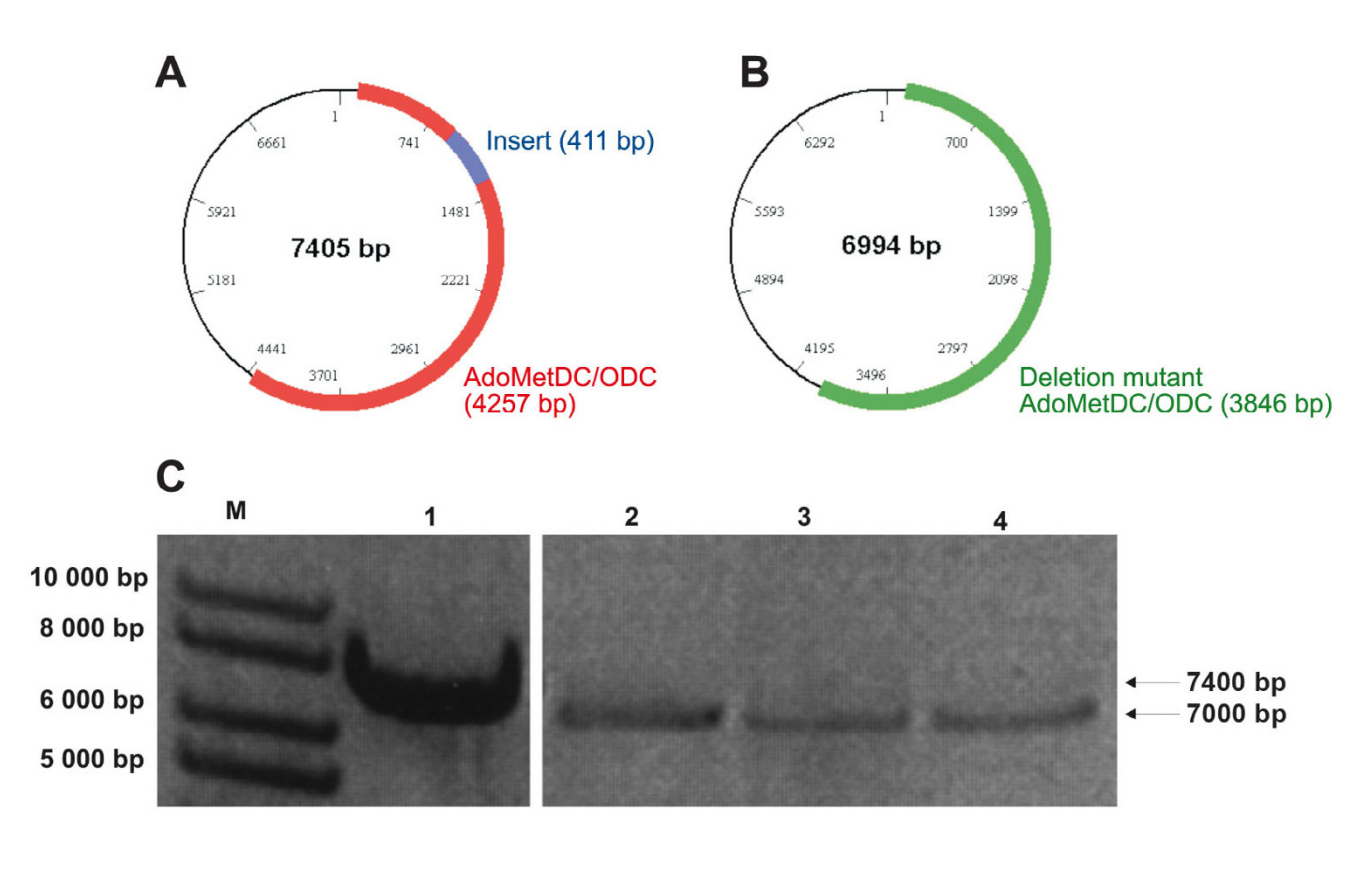
**Agarose gel electrophoresis of deletion mutant AdoMetDC/ODC PCR products**. Schematic representations of the wild type (A) (gene size 4257 bp) and the 411 bp deletion mutant (B) (gene size 3846 bp) AdoMetDC/ODC genes inserted into a pASK-IBA3 vector (vector size ~3100 bp). Wild type AdoMetDC/ODC is shown in red, the insert region is in blue, and the deletion mutant AdoMetDC is shown in green. The agarose electrophoresis gel of the deletion mutagenesis PCR products is given in (C) indicating the wild type PCR product of ~7400 bp and the deletion mutants of ~7000 bp produced by the different deletion mutagenesis methods. M) 1 kb marker, 1) wt AdoMetDC/ODC, 2) Overlapping primer PCR product, 3) Inverse PCR product, and 4) RE-mediated inverse PCR product.

**Figure 4:**
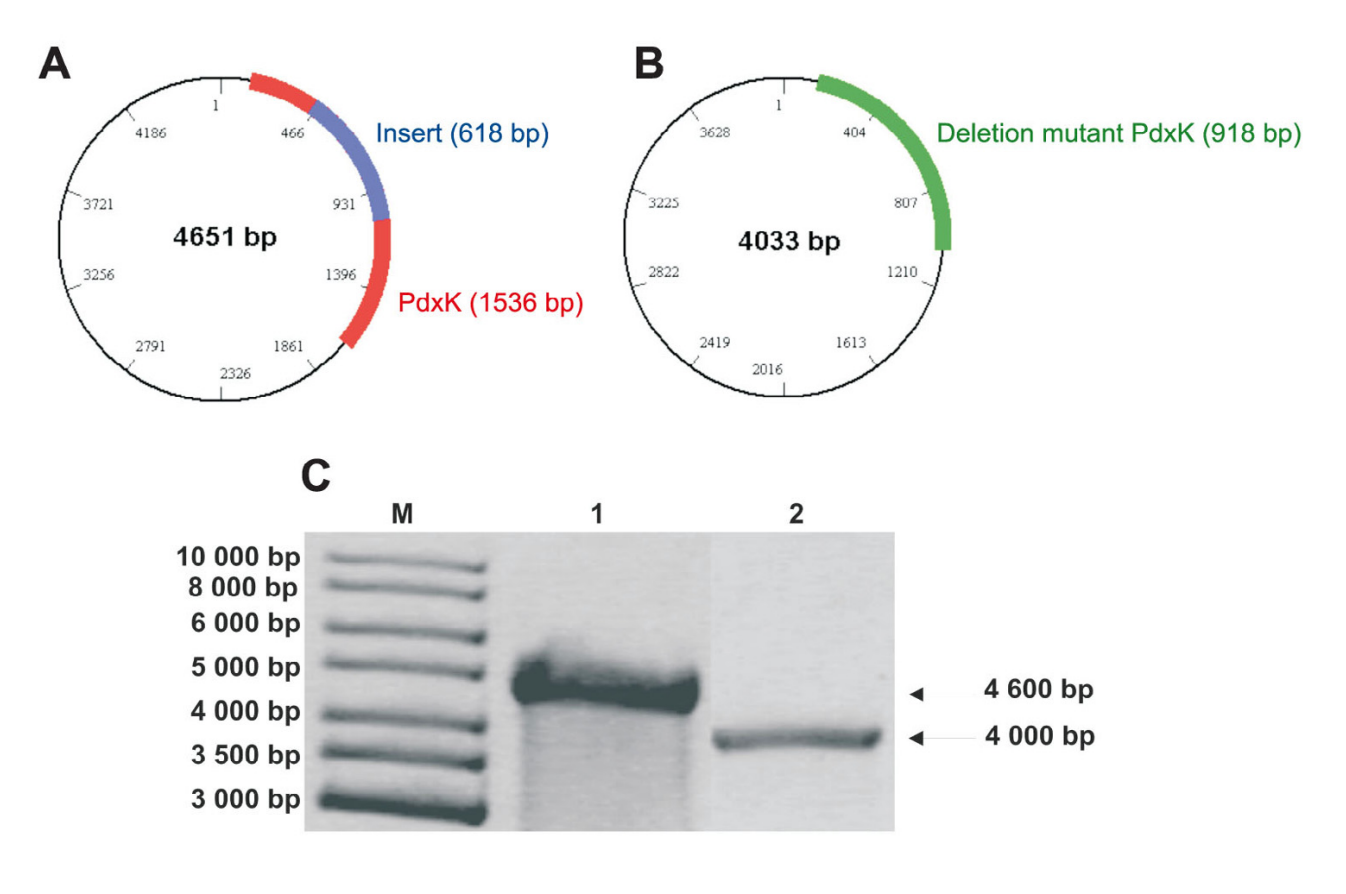
**Agarose gel electrophoresis of deletion mutant PdxK PCR products**. Schematic representations of the wild type (A) (gene size 1536 bp) and the 618 bp deletion mutant (B) (gene size 918 bp) PdxK genes inserted into a pASK-IBA3 vector (vector size ~3100 bp). Wild type PdxK is shown in red while the deletion mutant PdxK is in green. The agarose electrophoresis gel of the deletion mutagenesis PCR product is given in (C) indicating the wild type product of ~4600 bp and the deletion mutant at ~4000 bp produced by the RE-mediated inverse PCR method. M) 1 kb marker, 1) wt PdxK, 2) RE-mediated inverse PCR product.

The wild type malarial AdoMetDC/ODC cloned into pASK-IBA3 was used as template for a comparative study of all the deletion mutagenesis methods described here (Figure 3, lane 1). Subsequent gel electrophoresis analysis of the PCR products obtained by the different methods showed that only the overlapping primer method, inverse PCR method and RE-mediated inverse PCR method yielded products of the expected ~7 kb size for the deletion mutants (Figure 3). However, no PCR products could be visualised for the QCM or ExSite™ methods. The products obtained by the overlapping primer method, inverse PCR and RE-mediated inverse PCR method were further analysed for mutagenesis efficiency by *Hind*III restriction and occurrence of the expected ~3900 and ~3100 bp bands (Table 2). Two out of the five clones obtained by the overlapping method were mutant and did not contain the ~400 bp insert. No transformation-competent mutated genes were obtained for the inverse PCR method. The RE-mediated inverse PCR method described here resulted in four mutated out of five clones analyzed. Nucleotide sequencing verified the 40% and 80% mutagenesis efficiencies obtained for the overlapping primer and RE-mediated inverse PCR methods, respectively (Table 2). The subsequent application of the RE-mediated inverse PCR method on a second gene resulted in the deletion of a 618 bp PfPdxK parasite-specific insert as visualized by agarose electrophoresis (Figure 4C) and resulting in five out of five mutant clones (100%) (Table 2).

**Table 2:** Deletion mutagenesis efficiency results for the different protocols used. Five clones were analysed for each of the different PCR-based mutagenesis methods based on duplicate PCR experiments.

**Primer pair**	**Mutagenesis method**	**PCR product analysed with agarose gel electrophoresis**	**Restriction enzyme mapping with *Hind*III**	**Deletion efficiency confirmed with nucleotide sequencing (%)**
P1	QuickChange™ site-directed method	No product	NA	0
P2	Overlapping primer method	7 kb	~3900 bp ~3100 bp	40
P3	ExSite™ method	No product	NA	0
	Inverse PCR method	7 kb	~3900 bp 3100 bp	0
	RE-mediated inverse PCR method	7 kb	~3900 bp ~3100 bp	80
P4	RE-mediated inverse PCR method	4.6 kb	*Eco*RI linearization site removed	100

## Discussion

Due to the ever-increasing resistance of malarial parasites to commercially available drugs, it is of extreme importance to identify novel drug targets. Studies of the essential *P. falciparum *AdoMetDC/ODC bifunctional protein have led to the development of the RE-mediated inverse PCR method reported here. This enabled investigations of the structure-activity relationships of the large parasite-specific inserts of this bifunctional protein.

The RE-mediated inverse PCR method was compared to four other widely used PCR-based mutagenesis methods. The QuickChange™ site-directed mutagenesis method (QCM) requires that both of the mutagenic primers contain the desired mutation and anneal to the same sequence on opposite strands of the plasmid. The method is limited to primers of 25 to 45 nt in length with melting temperatures approximately 10°C above the extension temperature of 68°C. The mutation should preferably be in the centre of the primer flanked by at least 10 bases on either side. The GC content must also be at least 40%, and the primers must terminate in a G or a C base, which is difficult when working with the A+T-rich genome of *P. falciparum*. The QCM method claims 80% efficiency for point-mutagenesis but was unsuccessful in this deletion mutagenesis study. This also explains the inconsistent results produced in this laboratory by application of the QCM deletion mutagenesis method for the deletion of significantly sized areas in various other malaria genes [11].

The ExSite™ PCR-based site-directed mutagenesis method uses higher template concentrations and reduced PCR cycles to minimize potential second-site mutations. Primers for this method must be greater than 20 bases in length. The mismatched portions of the primers should be at or near the 5'-terminus of one or both of the primers with 15 or more of the matching sequence at the 3'-terminus. One or both of the primers must be 5' phosphorylated. In order to make a specific mutation, the alteration must be included within the primers and their 5'-termini must meet but not overlap. Any bases between the 5'-termini will subsequently be deleted in the final product. The application of the ExSite™ method did not result in any product.

Zheng and his co-workers modified the QCM protocol by using primers with partial overlaps at the 5'-termini to prevent self-extension (overlapping primer method, [19]). This method was applied to vectors of up to 12 kb in length and yielded significantly improved PCR mutagenesis results. The modified primers were proposed to overcome the limitation of the melting temperature of primer design dictated by QCM. At least 8 non-overlapping bases must be present on the 3'-termini of the primers, and the mutations may be as close as 4 nt away from the 5'-terminus. The primers must also terminate in a G or a C residue. The overlapping primer method was reported to delete up to 7 bp [19] and had a 40% mutagenesis efficiency in deleting 411 bp from the ~7.4 kb template in this study.

Inverse PCR employs two inverted tail-to-tail primers to amplify an entire gene/vector except for the region that needs to be deleted. This method has been successful in deleting up to 102 bp in large plasmids (12 kb) [18]. According to Wang *et al.*, for this method to be effective the primers must be similar in size, between 30 and 45 nt in length and have a melting temperature of at least 78°C with an applied annealing temperature of 68°C [18]. The number of PCR cycles must also be preferably less than 20. The application of this method produced the expected 7 kb deletion product as judged by gel electrophoresis only in the absence of DMSO. However, the correct deletion product was not present in any of the 5 clones screened (Figure 3, lane 3). The primary causes for such background after PCR-based mutagenesis techniques could include the mis-priming and subsequent generation of incorrect, transformation-competent PCR products or the self-annealing of the 5'-overhanging CGC ends of the primers that could prevent subsequent blunt-ended ligation [23]. The results presented here support other examples which suggests that a maximum of only 12 bp can be removed with the inverse PCR method [20].

**Figure 5:**
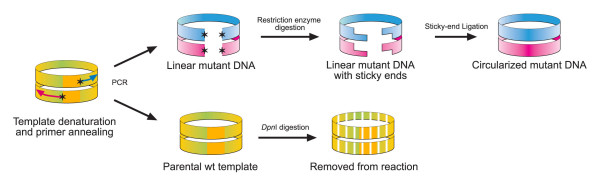
**Schematic representation of the RE-mediated inverse PCR method**. The RE-mediated inverse PCR reaction contains 15 pmol of both of the inverse primers (pink and blue arrows are the sense and antisense primers, respectively) and 0.01 pmol of the 7.4 kb template. The primers are designed in such a way that they contain unique restriction enzyme sites (represented by stars) and anneal to the opposite ends of the desired region to be deleted. The PCR cycle of template degradation for 45 sec at 95°C, primer annealing for 1 min at 56°C and primer extension for 2 min/kb at 68°C, is repeated for 18 cycles followed by a final extension step at 68°C for 2 min/kb. The PCR reaction results in the synthesis of both parental, wild type template DNA (yellow and green in the bottom panel), which is subsequently removed during a *Dpn*I digestion step, as well as linear mutated DNA (pink and blue in the top panel). Digestion with the unique restriction enzyme creates linear DNA with sticky-ends, which improves the ligation efficiency and subsequent circularization of the PCR product containing the deletion mutation.

The inverse PCR method was subsequently modified to incorporate unique restriction enzyme sites at the 5'-ends of both the sense and antisense inverted tail-to-tail primers (RE-mediated inverse PCR, Figure 5). The primers of the RE-mediated inverse PCR method designed here included 5'-terminal overhangs (CGC in this instance) to improve the efficiency of the restriction digestions. This is followed by unique restriction enzyme sites that generate sticky-ends to improve the ligation efficiency [24]. This would additionally increase the number of deletion products by eliminating any primary product still containing the inserted region. The designed primers were not dependent on a similar length due to the requirement to terminate in one or more G or C bases at the 3'-end to increase the specificity of the PCR reaction. This feature is particularly important in the application of PCR on A+T-rich *P. falciparum *genes. This method produced four out of five correct mutated products for a large area (411 bp) in a large gene (PfAdoMetDC/ODC, gene size 4257 bp). Additionally, this method proved efficient in deleting up to 618 bp in a smaller *P. falciparum *gene (PfPdxK, gene size 1536 bp). This indicates that the efficacy of this method may be influenced by the template size, with smaller templates resulting in marginally higher efficacy. However, this is not dependent on the particular gene sequence.

The RE-mediated inverse PCR method is a straightforward method in which the primers do not require either 5' phosphorylation or purification by PAGE or HPLC as specified by the general inverse PCR protocol [18]. Large deletions can be made without increasing the length of the primers as the desired mutation is not incorporated into the primer sequence itself but is simply deleted by extending the plasmid during the PCR reaction. A further advantage of this method is that the PCR temperature cycles of less than 20 are needed, which decreases the incidence of DNA polymerase error rates. The method was not dependent on the addition of 5% DMSO (results not shown) as is often needed by the inverse PCR method for the prevention of secondary structure formation in both primers and template. Additionally, there is no requirement for a high primer GC content as with the QCM method, which again is useful with the A+T-rich *P. falciparum *genome. Primer options for QCM and inverse PCR are furthermore limited by the fact that the melting temperature of the primers must be ≥ 78°C, which was not the case in the RE-mediated inverse method.

## Conclusion

The inability in deleting a reasonably large (>100 bp) DNA region with existing oligonucleotide-based deletion mutagenesis methods led to the application of a highly efficient RE-mediated inverse PCR method for the deletion of large areas in abnormally large *P. falciparum *genes. The method incorporates unique restriction enzyme sites at the 5'-ends of inverse tail-to-tail primers. In the absence of unique restriction sites, alternative methods including DiSec/TriSec [25], which allows the generation of specified sticky-ends, may be used or a restriction-independent method like the overlapping primer method should suffice. The method has proven to be invaluable in deciphering the involvement of parasite-specific inserts in structure-activity relationships of PfAdoMetDC/ODC and PfPdxK (manuscript in preparation).

## Authors' contributions

MW performed all the experiments and drafted the manuscript. AIL conceived the idea and LB designed the experiments and all three authors contributed equally in finalising the manuscript.
